# Online in-class vs. out-of-class flipped learning models in English as foreign language writing classes

**DOI:** 10.3389/fpsyg.2022.1009800

**Published:** 2022-11-14

**Authors:** Fatma Sengul, Hanife Bensen Bostanci, Mustafa Kurt

**Affiliations:** ^1^Department of English Language Teaching, Near East University, Nicosia, Cyprus; ^2^Research Center for Applied Linguistics (RCAL), Nicosia, Cyprus

**Keywords:** flipped classroom, in-class writing, out-of-class writing, traditional model of online writing, English as a foreign language learners, writing, perceptions

## Abstract

Flipped learning models are considered as important elements of English as a foreign language (EFL) writing courses in order to advance the EFL learners' writing skills. Significantly, studies examining the efficacy of in-class and out-of-class writing models in flipped classroom settings when teaching online EFL writing courses are still of focus in the Turkish Cypriot context. This investigation aimed to examine the most efficient flipped learning model among the in-class vs. out-of-class writing models for the purpose of helping instructors to advance their EFL learners' writing achievement in an online writing setting. In addition, this study sorted to reveal the EFL learners' perceptions toward learning writing through in-class and out-of-class flipped learning writing models. A mixed methods research design was applied to achieve the aforementioned aims. Twenty-eight EFL learners studying at a private university's English Language Teaching department constituted the participants of this study. As the findings pointed out, the EFL learners in group A who wrote their essays in-class outperformed those in group B, who wrote their essays out-of-class. Moreover, it was found that the majority of the participants had more positive perceptions toward the in-class flipped classroom writing model. This study highlights that, better learner performances are achieved when the learners write during the class session online with the support of the instructor when implementing a flipped classroom model to teach EFL writing.

## Introduction

Focusing on ways to improve English as a Foreign Language (EFL) learner's writing skill has been of focus, as writing is regarded as the most important skill that cannot be left behind in the instructional process (Khudhair, 2016). Writing is perceived of as a tool of creation that enables human beings to convey their ideas interactively for communicative goals as only with the help of writing can human beings transmit their ideas to a great number of people (Koura and Zahran, 2017). Although writing is an indispensable part of the instructional process, it is the most complex skill, which is challenging to both learn and teach (Bukhari, 2016). Taking these aspects into account, a great deal of importance is given to enhancing the writing skills of the EFL learners in the North Cyprus context, as writing is considered as a fundamental skill that should be taken into consideration more than the other skills, with a view to having a better academic and professional life (Brooks, 2012; Bostanci and Çavuşoglu, 2018). In North Cyprus, EFL learners study a year of compulsory English at a preparatory school, where they take specific classes in English and continuously write in English, with the objective of boosting their academic literacy skills and meeting future personal and public expectations, depending on their language levels, as well as in line with the major subject they study (Turgut and Kayaoglu, 2015). On top of these, universities begin to hire qualified lecturers, lessen the number of learners in the classes and begin to embed technology in the instructional process with the goal of increasing the quality of education (Shapiro, 2015). It has also been found that recent technological developments have enabled new means of teaching by providing learners a comfortable, enjoyable, motivating and relaxing instructional atmosphere (Bishop and Verleger, 2013; Ekmekçi, 2017).

The implementation of a new technological innovation or developments in the field of EFL education could be carried out with the help of the flipped learning model. It is commonly used nowadays and is considered as an alternative way of teaching where learners are autonomous and responsible for their educational process (Abaeian and Samadi, 2016; Aidinlou et al., 2017; Alastuey and Galar, 2017; Ekmekçi, 2017). Primarily, flipped learning plays a significant role in the instructional process, as it enables the learners to analyse the course content even before attending the lesson through the presentation of instructional videos about the course content. This step is followed by activities that check the level of understanding of the learners regarding the course content. This model advances the efficient and common usage of technological innovations in an out-of-the-class environment, so as to fulfill the instructional objectives (El-Sawy, 2018). As a result, the writing process becomes even more individualized by the use of the flipped learning method (Ekmekçi, 2017). A flipped learning writing model could be an influential method of instruction to advance the writing achievement level of EFL learners, particularly, in the Turkish Cypriot context (Ekmekçi, 2017; Soltanpour and Valizadeh, 2017; Salem, 2018). The traditional model of flipped learning in an online writing lecture is considered as out-of-class writing, where the learners write their essays out of the lecture and produce their essays at their own pace (Ahmed and Asiksoy, 2018; Altas and Mede, 2021). Another flipped model of teaching writing is in-class writing, where the learners write in a class with the instructor's guidance and where the teachers have more chance to embolden individual learning, provide ontime feedback, correct the learners' misconceptions and help the learners to practice their recent knowledge (Ping et al., 2019).

This study aimed to examine the most effective FLM for EFL learners of higher education by focalizing on the impacts of two different flipped learning models (FLMs), namely, in-class and out-of-class writing models. It further aimed at revealing the EFL learners' perceptions toward the implementation of these two models.

To achieve these objectives, the following questions were asked with a view to be answered:

1) Is there any significant difference among the EFL learners' writing performances, who were lectured with the in-class vs. out-of-class flipped learning models (FLMs)?
2) What are EFL learners' perceptions and preferences toward the in-class and out-of-class flipped learning models (FLMs) in writing courses?

## Literature review

During the implementation of English as a foreign language (EFL) education process, among the four skills, namely, speaking, reading, listening, and writing, writing is perceived to be one of the major influential skills. A great amount of attention is paid to improve the writing skills of the learners, to enable them to reach a requisite proficiency level in written communication in the Turkish Cypriot context (Ekmekçi, 2017). In general, teaching writing in the EFL context is regarded as a challenging task, as it is not easy to form, organize and interpret the thoughts through meaningful messages (Al-Shaer, 2014; Suyanto, 2015; Febrijanto, 2016; Ningrum et al., 2016; Koura and Zahran, 2017). Nonetheless, there are a great deal of issues in EFL writing lectures, specifically in the North Cyprus context (Afrilyasanti et al., 2016; Koura and Zahran, 2017; Dimililer and Kurt, 2019). In the same way, another problem that can be observed in the writing process in this specific context pertains to time constraints, as the lecturers have to spend a majority of the lecture time to explain the instructions before beginning the writing task, thus, there is limited time left for the writing process itself (Bostanci and Sengul, 2018). The writing process is perceived as challenging, complex and problematic for the EFL learners (Mahnam and Nejadansari, 2012; Bostanci and Çavuşoglu, 2018). Considering these aspects, the lecturers should be encouraged to renew their educational methodologies and techniques, to decrease the number of problems and difficulties that may arise and should do their best to convert such learning sessions into more inspirational, motivating, enjoyable, and self-sufficient classes (Ekmekçi, 2017).

Technology-oriented educational methods and models are seen to boost EFL learners' active participation and attendance in the educational process, and advance their language learning experiences (Salem, 2018). One way of implementing recent technological innovations in the educational process can be realized through flipped learning, which is a method that has gained attention in North Cyprus (Fraga and Harmon, 2014; Bostanci and Sengul, 2018; Salem, 2018). Flipped learning is an instructional methodology that puts emphasis on learner-oriented education and plays a significant role among many lecturers and researchers, all over the world (Güvenç, 2018). As the traditional flipped learning model is restricted with online video lectures and increased class time for in-class tasks and decreased time for in-class writing, it has been improved to authorize for several techniques and strategies that are convenient and suited to the requirements of separate fields, units, courses, and lectures (Kim, 2017). Thence, it creates a more comfortable and adaptable instructional atmosphere, as the EFL learners can acquire the information everywhere and every time they wish (Ekmekçi, 2017). The findings of flipped learning studies showed that flipped learning has the capability to assist instructors to advance their educational outcomes in the technology-integrated lecture (Aidinlou et al., 2017). Without any doubt, there is a substantial difference in the educational outcomes of the EFL learners who were lectured on flipped learning models (FLMs) and traditional educational strategies. It was also discovered that EFL learners who were taught in the flipped classroom-based lectures received higher grades than the EFL learners who were taught in traditional classes (Aidinlou et al., 2017). To summarize, utilizing flipped learning models (FLMs) in EFL classes increases learner outcomes (Suranakkharin, 2017).

In terms of learner preferences, learners preferred to be educated in a flipped learning-based environment, as it provides them the chance to take the authority of their personal learning process (Fraga and Harmon, 2014). Evidently, both the lecturers and EFL learners' attitudes toward the flipped learning process are positive (Alastuey and Galar, 2017). Flipped learning models (FLMs) can be considered as today's most appropriate instructional method for teaching writing to EFL learners (Hsieh et al., 2017; Güvenç, 2018; Öznacar et al., 2019). Flipped learning models in the writing process can be categorized into in-class and out-of-class that can be employed to respond to several pedagogical objectives (AbuSeileek and Qatawneh, 2013; Thakare, 2018).

Indeed, in-class writing enables the EFL learners to focus more on the writing process, organization of the essay and the thesis statement, while it pays less attention to the lexical items and mechanics of the language (Saed and Ghazali, 2017). Significantly, simultaneous learner and instruction presence is a must, as it fosters deeper and meaningful language learning with the presence of the instructor, peers and collaborative environment (Oztok et al., 2013; Perveen, 2016). In other words, the writing process occurs on time and it is considered as more expedient which enables the learners to be present in the classroom as “real” and “there” where the learners are able to reach a mutual understanding (Oztok et al., 2013; Lowenthal et al., 2017). Particularly, in-class writing enables the learners to feel the sense of community, and it also provides ontime feedback which advances the level of motivation and keeps the learners engaged in the activities as a result of the instructor's and other learners' presence (Perveen, 2016). Furthermore, it contributes to the cognitive presence and the learners' positive attitudes toward the EFL learning process (Oztok et al., 2013).

In contrast to these, a majority of the learners avoid attending and participating in the classroom environment at a particular hour, and some of them have problems regarding technology and network connection, and some of them also have issues regarding the scheduling and time zone (Oztok et al., 2013; Lowenthal et al., 2017). Additionally, as it is agreed, there is less time to think and produce an essay in the classroom environment, as well as restricting the usage of outside-of-class resources (AbuSeileek and Qatawneh, 2013). As it is claimed, in-class writing can be supported with the help of facilities and materials such as discussion rooms, real-time chat or online meeting platforms (Huang and Hsiao, 2012). Conversely, as the stated issues and the lecture time are limited, some of the lecturers prefer to ask their learners to write their essays out of the instructional environment, as the majority of lecture time is spent in describing the explanations and instructions about the writing task, rather than giving time to the EFL learners to practice their writing skills (Chang, 2016; Lin and Hwang, 2018). Out-of-class writing can be reached at every time and everywhere, and enables reflection, critical thinking and deeper learning (Oztok et al., 2013; Lowenthal et al., 2017). As it happens at a delayed time, it does not enable real-time access for instructional objectives, whereas the time of learning depends on the learner preferences, which help the instructors to avoid any misbehavior in the classroom environment and embolden the multiple learning styles (Oztok et al., 2013). As it is believed, out-of-class writing is mainly preferred for reflective and higher order thinking activities. It also supports the constructivist-oriented instruction, as it enables the learners to log in and communicate whenever they want, depending on the time that is convenient to them. It also gives them an opportunity to learn and work at their own pace within a scheduled time frame, to think, respond, plan and use external resources (AbuSeileek and Qatawneh, 2013; Oztok et al., 2013; Lowenthal et al., 2017; Karaaslan et al., 2018). In addition, an out-of-class writing model can be supported with e-mails, discussion platforms, blogs, audio-, and video recordings and so on (Huang and Hsiao, 2012).

As it is underlined in the study of AbuSeileek and Qatawneh (2013), both in-class and out-of-class writing models enable the learners to increase their writing performances. In a similar manner, Shang (2017) found that, both in-class and out-of-class writing had a positive effect on the EFL learners' writing abilities. Lowenthal and Dunlap (2017) suggested that, both in-class and out-of-class writing models should be used in writing classes, as they provide different advantages and disadvantages and affect the writing process in a different way. On top of these, although both in-class and out-of-class writing models help the learners to advance their writing skills, Saed and Ghazali (2017) proved that EFL learners had more positive attitudes toward the in-class writing model. As a result, as it is highlighted in the study of Oztok et al. (2013), the majority of their EFL learners prefer to write in-class, as it gives them more chance to collaborate with the class members and the instructor while writing. In contrast to these, the recent study of Bailey et al.'s (2020) indicated that that out-of-class writing had a positive impact on the learners' language skills.

Overall, both the in-class and out-of-class flipped classroom models maintain lifelong learning abilities and meaningful knowledge, rather than demanding the learners to memorize the knowledge for a specific time, and the instructors begin to instruct how to acquire the information besides only teaching the content in North Cyprus (Ahmed and Asiksoy, 2018; Sengul and Bostanci, 2021). Crucially, numerous investigators have paid attention to the flipped classroom, learner perceptions and different subject areas; however, none of the researchers have put emphasis on the effects of the in-class and out-of-class flipped classroom models on the EFL learners' writing improvement that fulfill the twenty-first century EFL learners' requirements by giving them a chance to engage in the tasks that help them to advance their abilities, such as critical thinking, problem solving, creative learning and communication (Ahmed, 2016; Sengul and Bostanci, 2021). More than these, EFL instructors faced with difficulties and failed to have an effective writing course based on online flipped classroom, as there is no study that had investigated the most appropriate and effective flipped classroom models among the in-class and out-of-class flipped classroom models for online writing course regarding the EFL learners' academic achievement in writing process at university-level EFL learning in North Cyprus (Ahmed, 2016; Sengul and Bostanci, 2021). In fact, this investigation is different from the previous researches, in that it purposed to flip EFL writing classes with different online flipped classroom models and encouraged courses with different flipped classroom formats and fill in the gaps regarding the implementation of different flipped classroom models, namely, in-class and out-of-class writing in online EFL writing courses at North Cyprus. Consequently, this study is innovative, as it provides a framework on how to design the writing courses based on the most convenient flipped classroom models in the online EFL writing context. It is essential, as it might encourage instructors and university personnel to implement the most appropriate flipped classroom model in EFL courses at North Cyprus and in the worldwide context to have a beneficial and effective online writing course.

## Methodology

### Research design and procedures

A mixed methods research design was implemented to effectively answer the research questions posed. Mixed methods employ both qualitative and quantitative data analysis and collection processes (Green, 2015).

Primarily, the investigation paid particular attention to the fourth-year English language (EFL) learners' written text analysis, to examine the impacts of the in-class vs. out-of-class flipped learning models (FLMs) on their writing performances. In both groups, where the in-class or out-of-class flipped classroom models were implemented, the same strategies, techniques, and procedures were utilized. The only difference was that one group wrote in a class hour with the help of the lecturer and the other group wrote at their own time and pace.

The following steps and procedures were employed. First, all the participant EFL learners in the class were asked to review the provided information and perform the provided activities related to each week's subject from the private university's flipped learning platform (online), before attending the online class through Google Meet. During the online class hour, all the participants completed the provided exercises related to each week's subject on Saturday's and after these processes, half of the learners (Group A) were asked to write their essays in-class, during an online class hour on Tuesdays and the other half of the learners were asked to write their essays out-of-class, whenever they preferred. Following this, all the participants sent their essays *via* e-mail to their peers (each week a different peer) for an indirect written corrective feedback and once they had made the necessary changes, the final essay was sent to the lecturer for lecturer's indirect written corrective feedback. The track changes and comments section on the Word processing document were implemented during the feedback process. Basically, the participants prepared two drafts before submitting their final products. The participants' final products were subjected to Turnitin plagiarism check before scores were set.

Learners were required to write five essay types in a period of 16 weeks including the midterm and final exam weeks and holidays. The essay types of the written texts could be arranged as follows: Task 1: Argumentative Essay, Task 2: Cause and Effect Essay, Task 3: Persuasive Essay, Task 4: Advantages and Disadvantages Essay, Task 5: Compare and Contrast Essay, midterm exam: Argumentative Essay and Cause and Effect Essay, and final exam: Persuasive Essay, Advantages and Disadvantages Essay, and Compare and Contrast Essay. Two weeks were provided for each essay type. Learners were required to write half of the essay in 1 week and the other half in the following week. Moreover, these written texts were graded out of 10 by the researcher and an ELT instructor, in line with the created essay writing criteria. In the examinations, two different essay types and two different essay topics for these types were presented for the participants to select. Learners had an hour's duration to complete the examinations online.

### Participants and sampling

Convenience sampling was employed, as the data were gathered among the EFL learners who were taking part in the course. Convenience sampling is a kind of non-probability sampling, which involves the participants from the target audience who fulfill specific convenient criteria, such as effortless reachability, geographic accessibility, accessibility at a particular time or the voluntariness to take part (Etikan et al., 2016). In particular, the participants were upper-intermediate level EFL learners who were taking the fourth-year writing course as an elective. They were divided into two groups as equally and randomly among the classroom members who were conveniently available to participate in the study without making any discrimination such as learning style, preference and so on.

The data were obtained during the 2021–2022 spring semester, from 28 EFL learners majoring in the Department of English Language Teaching at a private university, in North Cyprus. Fourteen of the participants formed Group A and 14 formed Group B. The participants in group A had an online writing lecture in a flipped learning writing class where the in-class writing model was utilized, whereas participants in group B had an online writing lecture in a flipped learning writing class where the out-of-class writing model was utilized.

### Data collection and analysis

**Figure 1:**
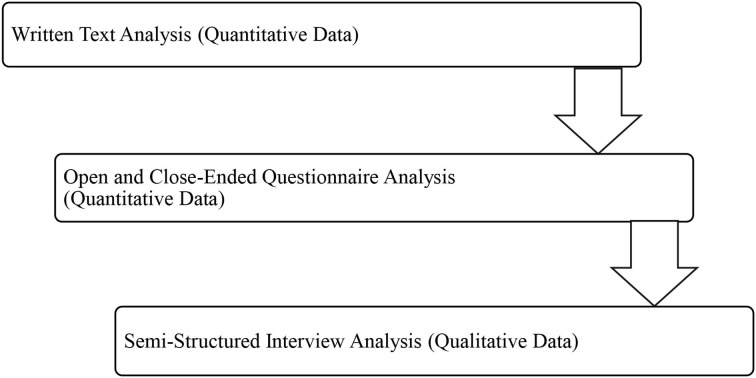
Phases of the mixed-methods.

Data were obtained both quantitatively and qualitatively. The quantitative data were obtained from the written essays (including the two examinations: midterm and final) of the EFL participants and a questionnaire that was employed after the treatment phase, whereas the qualitative data were obtained from the interviews carried out after the treatment phase (see Figure 1). The written text analysis was used to examine, whether there was an effect of the different flipped learning models (FLMs), namely, in-class vs. out-of-class writing, on the writing performance of the EFL learners. To do these, the written texts of the participants were stored in different folders for each week as Task 1, Task 2, Task 3, Task 4, Task 5, Midterm exam, and Final exam, then these written texts were scored out of 10 for each week. Following these, at the end of the term, a researcher-made questionnaire that consisted of both open and close-ended questions was filled in by the participants. Through the implementation of the open-ended questions, more information regarding the unique thoughts of the participants about the in-class vs. out-of-class flipped learning models (FLMs) was obtained. Close-ended questions were asked to collect generalized data about the participant perceptions toward the in-class vs. out-of-class writing in a flipped learning writing course (Jamshed, 2014). All the participants completed the questionnaire. Initially, the participants were asked about their background information such as age, gender, native language, country and years that they have been studying English by choosing the most convenient option. The questionnaire was applied through the use of Google Forms. In addition, to collect the qualitative data, the interview questions about the in-class vs. out-of-class flipped learning models (FLMs) for writing were asked to the participants during the online classroom hour through the use of the Google Meet online platform, and each group had a separate online meeting session for the interview process. The interview was applied to five EFL learners from group A and five EFL learners from group B in total, 10 of the participants volunteered to participate in the study. The semistructured interview included five open-ended questions in total, and the participants were asked about their beliefs and preferences regarding the implemented models through the use of a schematic presentation of questions. It took 10 min to interview each participant, in total it took almost 2 h to interview all the participants in both groups.

In brief, the following questions were asked as part of the interview:

1) What do you think about writing in-class and out-of-class in general? Is it essential or not? Is it beneficial or not to write in-class/out-of-class? Is it difficult or not? Why?
2) Do you use any resources while you write in-class/ out-of-class? If yes, what are these? Why do you prefer to use these sources?
3) Do you feel that you are improving your language proficiency in general (i.e., all skills, sub-skills and errors) or only in writing when you write in-class/ out-of-class? Why or why not?
4) If you had another chance to choose the environment that you will produce your written work from in-class or out-of-class, which one would you prefer? Why?
5) Is there anything that you want to add or share anything about writing in-class or out-of-class environment in the language learning process?

Data regarding the written texts were analyzed through the following steps. Initially, all the written texts were scored one by one, out of 10. Statistical Package of Social Sciences (SPSS) program version 20; descriptive statistics was employed to analyse the questionnaire data and essay scores. Henceforth, analysis of variance (ANOVA) test was used to crosscheck each group's participants' perceptions and preferences toward the in-class vs. out-of-class flipped learning models for writing. Furthermore, the key themes of each participant's interview transcript were separated to examine the reason behind the participants' positive and negative perceptions. Eventually, these key themes and patterns were defined, grouped and coded by the investigator.

### Validity and reliability

To ensure the reliability of the essay scores, the lecturer's and an ELT instructor's scores were set and compared employing a Pearson product-moment coefficient to find out the curvilinear relation among two variables. This has enabled the investigators to understand whether the existing correlation was condescending to the correct relation among these variables (Ravid, 2011). In the same way, the 2-tailed significance test was carried out to figure out if there was a positive or negative correlation among the variables and to see whether the relationship among these variables was statistically significant (see Table 1).

**Table 1:** In-class writing task 1.

		**Researcher's** **marks**	**Lecturer's** **marks**
Lecturer's mark	Pearson correlation	1	0.91^**^
	Sig. (2-tailed)		0.00
	*N*	14	14
Researcher's mark	Pearson correlation	0.91^**^	1
	Sig. (2-tailed)	0.00	
	*N*	14	14

** Correlation is significant at the 0.01 level (2-tailed).

As it is shown in Table 1, values under 0.50 are used to indicate the poor reliability whereas values between 0.50 and 0.75 indicate satisfactory reliability. Similarly, good reliability is shown between 0.75 and 0.90, whereas values over the 0.90 are used to emphasize the highest reliability value. So that, as the results pinpointed in Table 1, Pearson correlation value was 0.91, which means that the value of reliability is at the top point and there is a positive correlation between the lecturers' and English instructors' scores. In particular, there was no significant variation between the two raters' scores for the tasks, where the in-class writing was used for the writing process. In fact, the scores that were provided by both the investigator and instructor were reliable.

Moreover, the questionnaire, which was employed for the examination of the EFL learners' preferences toward the in-class vs. out-of-class flipped learning models for writing, was adopted and adapted from Sengül's (2018) questionnaire. To understand the efficacy of the questionnaire, a pilot study was carried out. The questionnaire was distributed to 30 EFL learners from the ELT department of a private university in Nicosia, during the 2019–2020 Fall semester. The findings of the pilot study were analyzed using Cronbach's alpha, to reveal the reliability of the questionnaire items (see Table 2). The findings revealed that the co-efficiency was 0.80, which refers to the fact that the questionnaire items were reliable (Virginia, 2015).

**Table 2:** Reliability statistics.

**CA**	**NI**
0.80	77

CA, Cronbach's alpha; NI, Number of items.

### Ethical consideration

Before carrying out the study, ethical approval was received from the Ethics Committee of the Institute of Educational Sciences of Near East University. Following approval, both written consent and oral consent were received by the participants. In the same way, the participants were also informed that the data collected for this study will be kept confidential and not accessed by anyone else, except the researchers. Pseudonyms were employed to hide the real identities of the participants.

## Findings and discussion

### Written text

To find out whether there was an impact of the different flipped learning models, namely, in-class and out-of-class writing on the EFL learners' writing performances and which flipped model advances the EFL learners' writing skills the most during the flipped writing process, this study compared the two groups.

#### In-class writing

The participants in group A, where the EFL participants produced their essays in the online class environment, were observed to have improved their writing skills after Task 1 (M: 3.43, SD: 1.01), as they had received better scores for Task 2 (M: 2.43, SD: 0.51). In Task 3 (M: 2.71, SD: 1.54), which was written as a midterm examination, their performance slightly decreased while they had improved their performance in Task 4 (M: 2.57, SD: 1.39). In the same way, they had improved their performance in Task 5 (M: 2.14, SD: 1.02), while their performance did not change in Task 6 (M: 2.14, SD: 1.02). Contrarily, the participants in group A increased their writing performance in Task 7 (M: 1.86, SD: 0.86), which was written as a final examination (see Table 3). These findings were similar to the findings of Saed and Ghazali (2017), who agreed that in-class writing had positive impacts on the EFL learners' writing skills. Apart from these, as the interview data presented the EFL participants in group A, namely, Janessa agreed that “In-class writing enables us to improve our four skills as we have a chance to collaborate with our classmates,” and Jack stated that “As we have a chance to receive instant feedback from the teacher and learn new vocabulary items from the discussions with our friends, in-class writing helps us to advance our speaking, listening and reading skills, in addition to our writing skills.” In other words, the reason behind the improvement of group A' s writing skills might be directly linked with the EFL learner's positive perceptions toward the in-class writing process, as it provided them more collaborative, engaging and motivated environment with the instructor's presence and on time feedback.

**Table 3:** The most effective flipped classroom model for writing.

**Tasks**	***N***	**In-class**		**Out-of-class**	
		**M**	**SD**	**M**	**SD**
1	14	3.43	1.01	4.79	2.08
2	14	2.43	0.51	3.57	1.34
3	14	2.71	1.54	4.21	1.84
4	14	2.57	1.39	3.50	1.55
5	14	2.14	1.02	4.43	1.86
6	14	2.14	1.02	4.21	1.67
7	14	1.86	0.86	3.93	1.32
Valid *N* (listwise)	14	

M, Mean score; SD, Standard deviation.

#### Out-of-class writing

The participants in group B where the EFL participants produced their essays out of the class environment were observed to have improved their writing skills after Task 1 (M: 4.79, SD: 2.08), as they received better scores in Task 2 (M: 3.57, SD: 1.34). However, the participants in group B decreased their performance in Task 3 (M: 4.21, SD: 1.84), which was written as a midterm exam, while their writing performance rapidly increased in Task 4 (M: 3.50, SD: 1.55). In comparison with these, the participants' writing performance rapidly decreased in Task 5 (M: 4.43, SD: 1.86). Nevertheless, the EFL participants' performance in Task 6 (M: 4.21, SD: 1.67) significantly improved. In the same way, the participants in group B increased their writing performances in Task 7 (M: 3.93, SD: 1.32), which was written as a final exam (see Table 3). These findings were in line with the Bailey et al.'s (2020) research findings, who agreed that out-of-class writing had a positive impact on the learners' language skills. With respect to the interview data, Angelina from group B stated that “Out-of-class writing enables us to advance our reading and listening skills in addition to our writing skills as we have to search for further information and materials related with the topic,” while Sue stated that “Out-of-class writing is not helpful at all, as during the in-class writing process the teacher's guidance enables us to notice our mistakes immediately and we can produce better essays with less mistakes.” Remarkably, it is not incorrect to claim that the EFL learners' writing performances in group B were influenced from the out-of-class writing process, as the participants need to search for further ideas and materials from the various sources without the instructors' ontime guidance and feedback.

Although the participants in both group A and group B improved their writing performances, it can be observed that the participants in group A received better scores than the participants in group B. In fact, the EFL participants in group A who wrote their written texts as in-class performed better than those in group B, who wrote their essays out-of-class. Similar to the findings of AbuSeileek and Qatawneh (2013) and Shang (2017) who found that, both out-of-class and in-class models helped their EFL learners to advance their writing skills, it was proved that in-class writing had a more positive impact on the learners' writing skills. To summarize, this study highlighted that in-class writing had more positive impact on the writing performances and achievements of the EFL learners in a flipped learning writing course, as it provided them more collaborative, engaging and motivated environment with the instructor's presence and ontime feedback.

### In-class vs. out-of-class FLM

Significantly, the participants were asked about their perceptions toward the in-class vs. out-of-class flipped learning models (FLMs) for writing, to reach more specific information. It was found that the participants in group A strongly agreed with the following statements: During the in-class writing process, we have more chance to get help from the teacher (M: 3.71, SD: 1.13), Writing out-of-class is time consuming (M: 3.64, SD: 1.49), I feel that I do not need to hurry up while I write out-of-class (M: 3.14, SD: 1.35), Getting into contact with our class mates for group work is much easier in the online environment (M: 3.07, SD: 1.26), and I feel more concentrated when I write in-class (M: 3.07, SD: 1.59). Moreover, they also agreed with the following statements: Both the pre-writing and writing process should be done in-class (M: 2.93, SD: 1.20), I prefer to write whenever I want (M: 2.93, SD: 1.26), I prefer to be taught in-class (M: 2.86, SD: 1.16), I can understand better what to do when the teacher guides me during the writing process (M: 2.79, SD: 1.31), I do not prefer to write out-of-class (M: 2.71, SD: 1.20), Teachers' guidance is necessary for better writing (M: 2.71, SD: 1.20), I write better when I collaborate with my class mates in-class (M: 2.71, SD: 1.20), I prefer to be taught out-of-class (M: 2.64, SD: 1.21), I can write better when I write without any time limitation (M: 2.64, SD: 1.27)I prefer to get help from friends (M: 2.71, SD: 1.13), I feel more concentrated when I write out-of-class (M: 2.64, SD: 1.39), I learn better when I write in-class (M: 2.50, SD: 1.01), and Online pre-writing exercises are more enjoyable (M: 2.50, SD: 1.01) (see Appendix Table 1).

On the other hand, the majority of the participants stated that they were neutral about the following statements: I feel that I need to hurry up while I write in-class (M: 2.43, SD: 1.34), I learn better when I write out-of-class (M: 2.36, SD: 1.39), In-class pre-writing exercises are more enjoyable (M: 2.36, SD: 1.33), I think it is more effective to write shortly after the pre-writing activities (M: 2.29, SD: 1.32), I write better when I collaborate with my class mates out-of-class (M: 2.21, SD: 1.12), I cannot concentrate on my writing at particular times of the day (M: 2.21, SD: 1.12), Both the pre-writing and writing process should be done out-of-class (M: 2.21, SD: 0.89), It is important to have an access to the online resources while writing (M: 2.21, SD: 0.97), Getting into contact with our class mates for group work is much easier in the classroom (M: 2.07, SD: 0.99), I prefer to get help from my lecturer (M: 2.07, SD: 1.20), and I prefer to use online sources when I do not understand something (M: 2.00, SD: 1.03). Contrarily, a great number of participants of the group A also disagreed with the following statements: I lose my concentration, when I write out-of-class (M: 1.93, SD: 0.91), During the out-of-class writing process we have more chance to get help from the teacher (M: 1.86, SD: 0.94), I lose my concentration, when I write in-class (M: 1.86, SD: 0.94), I do not prefer to write in-class (M: 1.64, SD: 0.84), and Writing in-class is time consuming (M: 1.64, SD: 0.84) (see Appendix Table 1).

As the results have indicated the majority of the EFL learner participants in group B strongly agreed with the following statements: I prefer to write whenever I want (M: 4.07, SD: 0.99), I can write better when I write without any time limitation (M: 3.86, SD: 0.94), Teachers' guidance is necessary for better writing (M: 3.71, SD: 0.99), I do not prefer to write out-of-class (M: 3.50, SD: 1.09), During the out-of-class writing process we have more chance to get help from the teacher (M: 3.50, SD: 1.01), I write better when I collaborate with my class mates out-of-class (M: 3.43, SD: 1.08), Getting into contact with our class mates for group work is much easier in the online environment (M: 3.21, SD: 1.67), I write better when I collaborate with my class mates in-class (M: 3.14, SD: 1.09), I learn better when I write in-class (M: 3.14, SD: 0.66), I feel more concentrated when I write in-class (M: 3.07, SD: 1.20), and I feel that I do not need to hurry up while I write out-of-class (M: 3.00, SD: 1.56). Furthermore, they also agreed with the following statements: I prefer to get help from friends (M: 2.79, SD: 1.05), Both the pre-writing and writing process should be done in-class (M: 2.79, SD: 0.97), During the in-class writing process we have more chance to get help from the teacher (M: 2.79, SD: 1.12), I feel more concentrated when I write out-of-class (M: 2.71, SD: 0.99), Writing in-class is time consuming (M: 2.71, SD: 0.72), Online pre-writing exercises are more enjoyable (M: 2.64, SD: 0.92), I learn better when I write out-of-class (M: 2.57, SD:1.15), and I prefer to be taught in-class (M: 2.57, SD: 0.75) (see Appendix Table 1).

Conversely, more than half of the participants in group B stated that they were neutral about the following statements: I think it is more effective to write shortly after the pre-writing activities (M: 2.21, SD: 0.89), In-class pre-writing exercises are more enjoyable (M: 2.14, SD: 1.23), I lose my concentration, when I write in-class (M: 2.14, 1.02), Both the pre-writing and writing process should be done out-of-class (M: 2.14, SD: 1.02), I can understand better what to do when the teacher guides me during the writing process (M: 2.07, SD: 0.99), Getting into contact with our class mates for group work is much easier in the classroom (M: 2.07, SD: 1.20), I cannot concentrate on my writing at particular times of the day (M: 2.00, SD: 0.87), and I prefer to use online sources when I do not understand something (M: 2.00, SD: 0.67). Significantly, majority of the participants also disagreed with the following statements: I lose my concentration, when I write out-of-class (M: 1.86, SD: 1.02), I feel that I need to hurry up while I write in-class (M: 1.71, SD: 1.13), I do not prefer to write in-class (M: 1.71, SD: 0.72), I prefer to get help from my lecturer (M: 1.64, SD: 1.08), Writing out-of-class is time consuming (M: 1.64, SD: 1.15), I prefer to be taught out-of-class (M: 1.50, SD: 0.65), and It is important to have an access to the online resources while writing (M: 1.43, SD: 0.64) (see Appendix Table 1).

To summarize, the findings of the research pinpointed that the EFL learner participants in groups A and B had positive perceptions toward the in-class writing, while they had negative perceptions toward the out-of-class writing. As the findings of the interview emphasized the EFL participants in group A, namely, Tom put forth that “In-class writing has a positive effect on our learning process as we have a chance to both make a research, and ask about the misunderstood issues to the teacher at the same time, which will help us to produce better essays,” and Diana agreed that “In-class writing is the best writing option for us as we can learn from our friends' mistakes and we have a more enjoyable and comfortable environment that we feel more motivated and concentrated to write.” Nevertheless, the participants in group B namely, Sue claimed that “In-class writing is better as it provides more supportive environment and through the collaborative activities we can brainstorm about the topic as a whole class besides of sitting alone and trying to find out new ideas to write about” and Michael stated that “As oppose to the out-of-class writing, in-class writing provides more supportive and creative environment that we can improve our speaking skills, while we are trying to reach further ideas about a particular subject to write about.” These findings were similar to the results of Huang and Hsiao (2012) and Karaaslan et al. (2018) who revealed that their learners had more positive perceptions toward in-class writing than out-of-class writing, as it provides more chances to increase the level of social interaction in the classroom environment. To put it simply, although the EFL learners believe that both in-class and out-of-class writing models have different positive and negative effects, they perceive in-class writing model to be more positively inclined during the flipped classroom-based online writing course, as it enables them to advance the collaboration, concentration, motivation, creativity and writing improvement as well as their speaking skills through the guidance of the instructor.

### Participant preferences

In order to find out a response to the primary research question regarding the EFL learners' flipped learning model preferences among in-class and out-of-class writing models, in the first section of the questionnaire, the participant EFL learners were asked questions about their in-class and out-of-class writing model preferences during the flipped learning writing process. The findings of the study showed that majority of the EFL learners in group A, where the writing process occurred in-class, favored in-class writing (M: 1.71, SD: 0.91). This finding could be supported with the interview data, where the participants in group A, namely, Betty stated that “I prefer to write in-class as it enables us to have a discussion with our friends and ask questions to our teacher about the misunderstood issues.” and Diana, put forward the claim that, “It is better to write in-class as we know that teacher will be always there for us.” On the other hand, a great number of the EFL learner participants in group B, where the writing process occurred as out-of-class, put forward the desire that they would prefer to have their writing classes both in-class and out-of-class (M: 2.21, SD: 0.89) during the flipped class-based writing process (see Table 4). In the same way, the participants in group B, namely, Hera strongly agreed that she would prefer to write in-class as “In-class writing provides more friendly, enjoyable, comfortable, and relaxed environment that advances our motivation and concentration,” whereas one of the participants in group B, Angelina stated that “It would be better to write as both in-class and out-of-class, as both of them have different advantages and disadvantages.” Significantly, these findings were similar to the findings of Oztok et al. (2013), who put forward the claim that a majority of the EFL learners favored in-class writing rather than out-of-class writing, as it provided more chance for social interaction and communication. On the contrary, the findings of Lowenthal and Dunlap (2017) revealed that a majority of their EFL learners believed that both in-class and out-of-class writing models should be implemented in the lectures, as they have different advantages and impacts on the learning process. As a result, it is not incorrect to claim that although both in-class and out-of-class writing models might have different advantages and disadvantages, the EFL learners had strong preferences toward the in-class writing model during the flipped classroom-based online writing, as it enabled them to advance their writing and speaking skills., It also helped in their motivation toward the writing process through the collaborative environment where the EFL learners had obtained a chance to work together in a friendly, enjoyable, comfortable, creative, and relaxed classroom atmosphere with the instructors' presence, on time guidance and feedback.

**Table 4:** English as a foreign language (EFL) learner's preference.

**Flipped learning model statement**	***N***	**In-class writing**		**Out-of-class writing**	
		**M**	**SD**	**M**	**SD**
If I had another chance to be taught writing, I prefer to write …	14	1.71	0.91	2.21	0.89
Valid *N* (listwise)	14		

M, Mean score; SD, Standard deviation.

## Conclusion

Overall, the research data regarding the initial research question which was purposed to analyse the most effective flipped classroom model for writing on the EFL learners' writing performances among the in-class and out-of-class writing models, the research data underlined that the most effective flipped classroom model for writing is the in-class writing model.

Following this, the results regarding the second question which purposed to analyse the EFL learners' perceptions toward the in-class and out-of-class writing models indicated that the majority of the EFL learners, who produced their written texts in-class, had positive perceptions toward the in-class writing model, while they had more negative perceptions toward the out-of-class writing model.

Lastly, as regards the EFL learners' preferences of a flipped learning model for writing among in-class vs. out-of-class writing models, the EFL learner participants, who produced their written texts in-class, favored in-class writing.

Consequently, it was highly suggested for pre-service and in-service instructors to implement the online in-class writing model into their flipped classroom-based writing courses, to create a more positive, collaborative and motivated environment. Through the implementation of online in-class writing models into the writing lessons, the instructors in university-level EFL learning would be better able to enable the EFL learners to improve their writing abilities and performances.

To conclude, this research has shed light on acquiring English as a foreign language writing skills to those instructors and researchers who would like to improve their EFL learners' writing abilities in a collaborative environment by implementing the necessary technology and also, by having a fruitful writing lesson in a technology-oriented environment. In fact, some recommendations were provided to help the researchers who would like to yield insights that would increase understanding in implementing more technology-oriented writing courses based on flipped classroom models. Significantly, this study is restricted to North Cyprus and if the context of the research differs, the findings might change. In addition to these, the designed questionnaire did not provide any information on the efficacy of the various flipped classroom models on the EFL learners' different skills such as reading, speaking and listening. Next, as the research was employed in a restricted time period the findings might vary, if the duration of data collection of the writing process got extended or vice versa. The findings might also vary due to the class size, level of the EFL learners and the number of participants who took part in it.

## Data availability statement

The original contributions presented in the study are included in the article/Supplementary material, further inquiries can be directed to the corresponding author.

## Ethics statement

The studies involving human participants were reviewed and approved by Ethical Committee Board of Near East University. The patients/participants provided their written informed consent to participate in this study.

## Author contributions

FS wrote the article. HB contributed to the conceptualization and the data collection process. MK helped to the generation of the methodology and supervised the article. All authors contributed to the article and approved the submitted version.

## Conflict of interest

The authors declare that the research was conducted in the absence of any commercial or financial relationships that could be construed as a potential conflict of interest.

## Publisher's note

All claims expressed in this article are solely those of the authors and do not necessarily represent those of their affiliated organizations, or those of the publisher, the editors and the reviewers. Any product that may be evaluated in this article, or claim that may be made by its manufacturer, is not guaranteed or endorsed by the publisher.
