# Supplementary material for: Online in-class vs. out-of-class flipped learning models in English as foreign language writing classes
Source: Front Psychol. 2022 Nov 14;13:1009800. doi: 10.3389/fpsyg.2022.1009800 (PMC9703047; doi:10.3389/fpsyg.2022.1009800)
Supplement: Supplementary file 1 [file Table_1.docx]

**SUPPLEMENTARY FILE**

**Appendix Table 1**

| *EFL Learners’ Perceptions* | | | | | | |  |  |  |
| --- | --- | --- | --- | --- | --- | --- | --- | --- | --- |
| **Statements about the flipped classroom models** | **N** | **In-Class Writing** | | **Out-of-Class Writing** | | | | |  |
|  |  | M | SD | M | | SD | | |  |
| 1. Writing out-of-class is time consuming. | 14 | 3.64 | 1.49 | 1.64 | | 1.15 | | |  |
| 1. Writing in-class is time consuming. | 14 | 1.64 | 0.84 | 2.71 | | 0.72 | | |  |
| 1. During the in-class writing process we have more chance to get help from the teacher. | 14 | 3.71 | 1.13 | 2.79 | | 1.12 | | |  |
| 1. During the out-of-class writing process we have more chance to get help from the teacher. | 14 | 1.86 | 0.94 | 3.50 | | 1.01 | | |  |
| 1. I prefer to get help from friends. | 14 | 2.71 | 1.13 | 2.79 | | 1.05 | | |  |
| 1. I prefer to get help from my instructor. | 14 | 2.07 | 1.20 | 1.64 | | 1.08 | | |  |
| 1. I prefer to use online sources when I do not understand something. | 14 | 2.00 | 1.03 | 2.00 | | 0.67 | | |  |
| 1. I learn better when I write in-class. | 14 | 2.50 | 1.01 | 3.14 | | 0.66 | | |  |
| 1. I learn better when I write out-of-class. | 14 | 2.36 | 1.39 | 2.57 | | 1.15 | | |  |
| 1. I think it is more effective to write shortly after the pre-writing activities. | 14 | 2.29 | 1.32 | 2.21 | | 0.89 | | |  |
| 1. I can understand better what to do when the teacher guides me during the writing process. | 14 | 2.79 | 1.31 | 2.07 | | 0.99 | | |  |
| 1. I prefer to be taught in-class. | 14 | 2.86 | 1.16 | 2.57 | | 0.75 | | |  |
| 1. I prefer to be taught out-of-class. | 14 | 2.64 | 1.21 | 1.50 | | 0.65 | | |  |
| 1. Both the pre-writing and writing process should be done in-class. | 14 | 2.93 | 1.20 | 2.79 | | 0.97 | | |  |
| 1. Both the pre-writing and writing process should be done out-of-class. | 14 | 2.21 | 0.89 | 2.14 | | 1.02 | | |  |
| 1. I feel that I need to hurry up while I write in-class. | 14 | 2.43 | 1.34 | 1.71 | | 1.13 | | |  |
| 1. I feel that I do not need to hurry up while I write out-of-class. | 14 | 3.14 | 1.35 | 3.00 | | 1.56 | | |  |
| 1. I feel more concentrated when I write in-class. 2. I feel more concentrated when I write out-of-class. 3. I lose my concentration, when I write in-class. 4. I lose my concentration, when I write out-of-class. 5. Online pre-writing exercises are more enjoyable. 6. In-class pre-writing exercises are more enjoyable. 7. Getting into contact with our class mates for group work is much easier in the online environment. 8. Getting into contact with our class mates for group work is much easier in the classroom. 9. Teachers’ guidance is necessary for better writing. 10. I write better when I collaborate with my class mates in-class. 11. I write better when I collaborate with my class mates out-of-class. 12. It is important to have an access to the online resources while writing. 13. I prefer to write whenever I want. 14. I do not prefer to write in-class. 15. I do not prefer to write out-of-class. 16. I cannot concentrate on my writing at particular times of the day. 17. I can write better when I write without any time limitation. | 14  14  14  14  14  14  14  14  14  14  14  14  14  14  14  14  14 | 3.07  2.64  1.86  1.93  2.50  2.36  3.07  2.07  2.71  2.71  2.21  2.21  2.93  1.64  2.71  2.21  2.64 | 1.59  1.39  0.94  0.91  1.01  1.33  1.26  0.99  1.20  1.20  1.12  0.97  1.26  0.84  1.20  1.12  1.27 | 3.07  2.71  2.14  1.86  2.64  2.14  3.21  2.07  3.71  3.14  3.43  1.43  4.07  1.71  3.50  2.00  3.86 | | 1.20  0.99  1.02  1.02  0.92  1.23  1.67  1.20  0.99  1.09  1.08  0.64  0.99  0.72  1.09  0.87  0.94 | | |  |
| Valid N (listwise) | 14 |  | |  |  | | |  | |

*Key: M: Mean Score SD: Standard Deviation*
